# Reviewed article: Siqueira PC, Sales CMM, Prado TN, Maciel ELN. Análise da completude das fichas de notificação de covid-19 na população indígena no Espírito Santo, 2020. Epidemiol Serv Saude. 2025:34;20240176

**DOI:** 10.1590/S2237-96222025v34e20240176.a

**Published:** 2025-04-14

**Authors:** Ana Carolina Micheletti Gomide Nogueira de Sá

**Affiliations:** Universidade de Minas Gerais

**Table:** 

Rating	Excellent	Good	Average	Below average	Poor
Interest		✓			
Quality			✓		
Originality			✓		
Overall			✓		

**Table:** 

Questionnaire	Yes	No	Not applicable
Does the manuscript contain new and significant information to justify publication?	✓		
Does the Abstract (Summary) clearly and accurately describe the content of the article?		✓	
Is the problem significant and concisely stated?		✓	
Are the methods described comprehensively?		✓	
Are the interpretations and conclusions justified by the results?		✓	
Is adequate reference made to other work in the field?	✓		

## Manuscript Structure:

Length of article is: Adequate

Number of tables is: Adequate

Number of figures is: Adequate

Please state any conflict(s) of interest that you have in relation to the review of this paper (state “none” if this is not applicable): Name

## Comments

A temática do artigo é extremamente relevante, principalmente em virtude da má qualidade de informações relacionados aos povos indígenas. Contudo, alguns aspectos chaves precisam ser trabalhados no artigo para que esteja apto para a publicação. Abaixo estão pontuadas algumas considerações:

1) O título, objetivo e métodos precisa de uma conexão. O título consta a palavra análise, no objetivo o verbo principal é avaliar e nos métodos os autores trazem como um estudo descritivo. É preciso alinhar esse conjunto. O estudo não é de avaliação. Não seria estimar a completude? Sugiro rever.

2) Não consta nos métodos do resumo o local do estudo, o período é a fonte de dados. Não fica claro para o leitor na conclusão do resumo, o que significa os achados variaram de excelente a muito ruim, o que foi melhor ou pior? O que mais chama a atenção? Sugiro ajustar.

3) Na introdução não constam dados epidemiológicos sobre o tema no mundo e no Brasil. A problemática do estudo não está clara. Em que este estudo contribui e avança em relação aos demais? Precisa rever. Além disso, ajustar o objetivo geral, conforme mencionado acima. Sugiro que desde o primeiro parágrafo já seja trazido o tema do estudo na população indígena para o leitor.

4) Nos métodos o estudo descreve que foi um estudo feito na cidade de Aracruz, por sua vez, em todo o texto dá a entender que foi para todo o Espírito Santo, precisa corrigir isso. Uma vez que dessa forma o estudo é representativo de Aracruz e não do ES. Rever. Outro aspecto é com relação ao tipo de estudo trata-se de um estudo transversal, isso não é mencionado. Pode-se acrescentar descritivo transversal. Além disso, os autores abordam ser um estudo descritivo, mas aparece que os dados foram avaliados com significância de 5% e não são apresentados testes estatísticos que levem a essa inferência. Precisa rever. Ademais, se foi utilizado significância de 5%, pressupõe-se que seria um estudo analítico, mas pelos dados da tabela 1 nota-se que é descritivo. Precisa ajustar isso. Sugiro utilizar e seguir o strobe.

5) Tabela 1: Precisa especificar as siglas, ajustar a localidade.

6) Na discussão, no segundo parágrafo repete resultado. Não constam limitações. Em todos os parágrafos da discussão precisa trazer maiores detalhes do que explicam os resultados do presente estudo.

6) As referências constam muitas literaturas cinzentas. Precisa equilibrar melhor com artigos.

